# Hepatotoxicity From Disease-Modifying Anti-rheumatic Drugs (DMARDs) and Association With Metabolic Syndrome in Rheumatoid Arthritis

**DOI:** 10.7759/cureus.78626

**Published:** 2025-02-06

**Authors:** Abhishek Nandan, Josna Haritha, David Maniscalco, Rabia Gill, Puneet Puri, Viviana Rodriguez, Huzaefah Syed

**Affiliations:** 1 Rheumatology, Virginia Commonwealth University School of Medicine, Richmond, USA; 2 Rheumatology, Hunter Holmes McGuire Veterans Affairs Medical Center, Richmond, USA; 3 Rheumatology, Virginia Commonwealth University, Richmond, USA; 4 Hepatology, Virginia Commonwealth University, Richmond, USA; 5 Biostatistics, Virginia Commonwealth University, Richmond, USA

**Keywords:** disease-modifying anti-rheumatic drugs (dmard), hepatic cirrhosis, hepatotoxicity, liver cirrhosis, liver enzymes, liver fibrosis, low-dose methotrexate, methotrexate, rheumatoid arthritis

## Abstract

Objectives: Hepatotoxicity is a frequent reason why disease-modifying anti-rheumatic drugs (DMARDs) are stopped or changed. We hypothesize that features of metabolic syndrome (such as obesity, dyslipidemia, and diabetes) are risk factors for hepatotoxicity leading to DMARD change in rheumatoid arthritis (RA).

Methods: We conducted a retrospective chart review of 361 patients with RA. Demographic information, lipid panels, smoking status, prior alcohol use, BMI, statin use, seropositive status, viral hepatitis serologies, and type of DMARD use were noted at the initial visit and at the time of DMARD change due to hepatotoxicity if applicable. Using exact logistic regression, odds ratios for the risk factors of DMARD change due to hepatotoxicity (primary outcome) were calculated.

Results: Twenty out of 361 patients with RA had their DMARD changed due to hepatotoxicity. Methotrexate (odds ratio {OR} 3.07) and leflunomide (OR 6.11) carried the highest OR for DMARD change. BMI > 35 (OR 2.14), diabetes mellitus II (OR 2.01), and alcohol abuse (OR 3.5) were associated with DMARD change due to hepatotoxicity but were not statistically significant. Rheumatoid factor or anti-CCP seropositivity did not appear to be associated with increased risk for the primary outcome.

Conclusion: Our study does suggest that several surrogates of metabolic syndrome may be associated with the risk of hepatotoxicity due to methotrexate and leflunomide. Though the study was underpowered to assess the risk for glycated hemoglobin (HbA1c) and obesity, these variables did trend toward increasing the risk. Clinicians should consider features of metabolic syndrome as potential risk factors for hepatotoxicity with DMARD use.

## Introduction

Hepatotoxicity is a common reason why disease-modifying antirheumatic drugs (DMARDs) are abandoned or changed in rheumatoid arthritis (RA). Routine liver biopsies have shown several hepatic changes ranging from steatosis to fibrosis in patients on DMARDs such as methotrexate and leflunomide [1-2]. However, many of these changes are non-specific and are also found in patients with metabolic syndrome [3,4]. While routine screening liver biopsies were once standard of care on patients with methotrexate, this is now no longer recommended for asymptomatic screening. Regular monitoring of transaminases has largely supplanted the need for routine liver biopsies. It is estimated that about 5% of patients on methotrexate will develop transaminase elevations that are twice the upper limit of normal [1]. Similarly, three-fold transaminase elevations have been noted in 7-13% of patients with leflunomide [5]. Persistent low-grade transaminase elevations or severe elevations are causes of concern for clinicians and patients who deal with these medications.

Therefore, attention has been drawn to the clinical significance of these transaminase elevations as well as their etiologies. Contrasted to psoriasis, patients with rheumatoid arthritis have a lower risk for methotrexate-associated hepatotoxicity, specifically in terms of hepatic fibrosis [2,3]. One hypothesis for such a finding has been the increased prevalence of non-alcoholic fatty liver disease/non-alcoholic steatohepatitis (NAFLD/NASH) in patients with psoriasis compared to those with rheumatoid arthritis. The prevalence of NAFLD in patients with psoriasis has been two to three times that of the general population in several studies [3], compared with rheumatoid arthritis and the general population which are largely no different [4].

NAFLD prevalence has been steadily increasing worldwide, with estimates worldwide having a median of twenty percent [5]. Insulin resistance which leads to increased hepatic uptake of triglycerides and hepatic fatty acids has been posited to play a key role in pathogenesis [6]. Metabolic syndrome features (obesity, hyperglycemia, dyslipidemia, and hypertension) have been showed to be strong predictors of NAFLD [7,8]. We hypothesize that features of metabolic syndrome (such as obesity, dyslipidemia, and diabetes) are risk factors for the development of hepatotoxicity with DMARD use in rheumatoid arthritis.

This article was previously presented as a meeting abstract at the 2018 American College of Rheumatology Annual Meeting on October 22, 2018.

## Materials and methods

We conducted a retrospective chart review of all patients who carried a diagnosis of rheumatoid arthritis on at least three consecutive clinic visits at our university medical center between January 2011 and December 2015. The study was approved by our university’s institutional review board (IRB# HM20006447). This retrospective search yielded a total of 361 patients for whom demographic information, BMI, lipid panel, prior alcohol use, smoking status, viral hepatitis serologies (for hepatitis B and C), statin use, rheumatoid arthritis seropositive status (based on the rheumatoid factor {RF} and anti-cyclic citrullinated peptide antibodies {CCP Ab}), and type of DMARD used were recorded at the initial visit to the rheumatology clinic. Alcohol use disorders were defined by provider documentation of quantity consumed, or secondarily, if available the provider documented judgment of abuse or appropriate use. If the exact quantity of alcohol consumption was documented, the use definition of abuse was > 7 drinks per week for women and > 14 drinks per week for men.

Inclusion criteria were age > 18, diagnosis of rheumatoid arthritis as noted above, at least three sets of liver function tests (including aspartate aminotransferase {AST}, alanine aminotransferase {ALT}, total/indirect bilirubin, and albumin), and being prescribed at least one DMARD. Exclusion criteria included another rheumatologic diagnosis (such as psoriatic arthritis), and pre-existing liver diseases other than NASH (such as hemochromatosis, alpha-antitrypsin 1 deficiency, or autoimmune hepatitis).

We subsequently went through each follow-up clinic note for the individual patients with a concerted effort (in combination with corresponding laboratory findings) to find if a DMARD was documented as changed or removed for the documented reason of hepatoxicity (primary outcome). Our rationale for this broad definition of the individual physician-led primary outcome is based on inconsistent guidelines from both drug manufacturers and professional societies on what exactly constitutes significant clinical hepatotoxicity. In clinical practice, definitions of liver function test elevations twice to five times the upper limit of normal are varyingly used as clinically significant hepatotoxicity. After data collection using exact logistic regression, odds ratios for the risk factors of DMARD hepatotoxicity were calculated. Methotrexate use, leflunomide use, statin use, alcohol abuse, a current statin, a BMI > 35, and diabetes mellitus II were used to calculate the adjusted odds ratios.

## Results

Our retrospective review yielded a total of 361 patients with rheumatoid arthritis who were reviewed. Baseline characteristics of the full cohort are recorded in Table 1 and Table 2. The patients were predominantly female individuals. There was a near-equal representation between patients of Caucasian and African American descent. Among the patients, 15.1% met the definition of Class II obesity and 17.8% carried a pre-existing diagnosis of diabetes mellitus II.

**Table 1:** Patient characteristics. ^a^Continuous variables presented as mean ± SD; ^b^N=320; ^c^N=315. EtOH: Ethanol.

Characteristic	Total N=361	
	n	%
Gender		
Female	297	82.3
Male	64	17.7
Race		
African American	170	47.1
Asian	7	1.9
Caucasian	172	47.6
-Other	12	3.3
Mean age at first visit^a^	52.5	13.5 (SD)
Mean age at last visit^a^	56.3	13.4 (SD)
BMI^a^	30.3	7.6
<25	99	31.2
25 - 35	170	53.6
≥35	48	15.1
Smoking status^b^		
Yes, current	78	24.4
Former	73	22.8
Never	169	52.8
Alcohol use^c^		
Active EtOH abuse	4	1.3
Previous EtOH abuse	11	3.5
Current	133	42.2
No EtOH use	167	53.0

**Table 2:** Clinical features closest to first rheumatology visit. ^a^Continuous variables presented as mean ± SD. VL, viral load; AST, aspartate aminotransferase; ALT, alanine aminotransferase; Hx, history; HbA1c, glycated hemoglobin, HDL, high-density lipoprotein; LDL, low-density lipoprotein; CCP, cyclic citrullinated peptide antibodies; RF, rheumatoid factor.

Characteristic	Total N=361		
	N	n	%
Hepatitis C status			
HepC AB+, +VL at diagnosis	236	11	4.7
HepC AB+, -VL at diagnosis	236	7	3.0
HepC AB+, unknown VL	236	0	0.0
HepC AB-	236	218	92.4
Hepatitis B status			
HepB sAg-	237	236	99.6
AST^a^	359	27.7	40.5
ALT^a^	359	27	26.6
Total bilirubin^a^	350	0.5	0.4
Albumin^a^	355	4.2	0.4
Current statin	346	73	21.1
Hx of diabetes mellitus II	343	61	17.8
HbA1c^a^	99	6.3	1.1
Triglycerides^a^	192	125.6	86.7
HDL^a^	194	53.7	17.4
LDL^a^	195	103.8	52.3
CCP positive	361	183	50.7
RF positive	347	180	51.9

Table 3 shows the DMARD initiated at the first visit in addition to the primary outcome, which is a physician-led change of DMARD due to concerns about hepatotoxicity. Most patients were started on methotrexate and/or hydroxychloroquine. Only a small minority of patients were started on other drugs including biologic therapies. AST and ALT were approximately twice to thrice the initial values upon provider-led DMARD change due to hepatotoxicity, the primary outcome.

**Table 3:** DMARD treatment. ^a^Continuous variables presented as mean ± SD; ^b^N=20. DMARD, disease-modifying anti-rheumatic drug; TNFi, tumor necrosis factor inhibitor; AST, aspartate aminotransferase; ALT, alanine aminotransferase.

Characteristic	n	%
DMARD started		
Methotrexate (MTX)	207	57.3
Leflunomide (Arava)	23	6.4
Sulfasalazine (SSZ)	46	12.7
Azathioprine (AZA, Imuran)	6	1.7
Tofacitinib (Xeljanz)	1	0.3
Tocilizumab (Actemra)	1	0.3
Hydroxychloroquine (HCQ, Plaquenil)	147	40.7
TNFi	49	13.6
Abatacept (Orencia)	6	1.7
Prednisone	127	35.2
Other	10	2.8
DMARD change or decrease in dose		
Unchanged or not decreased	154	42.7
Changed due to liver abnormality	17	4.7
Changed due to other reason	170	47.1
Same DMARD, but DOSE was decreased due to liver abnormality	3	0.8
Same DMARD, but DOSE was decreased due to other abnormality	17	4.7
AST after DMARD change^a,b^	77.8	47.5 (SD)
ALT after DMARD change^a,b^	90.0	44.36 (SD)
AST change (AST after DMARD - initial AST)^a,b^	45.2	43.0 (SD)
ALT change (ALT after DMARD - initial ALT)^a,b^	40.6	40.6 (SD)

**Figure 1:**
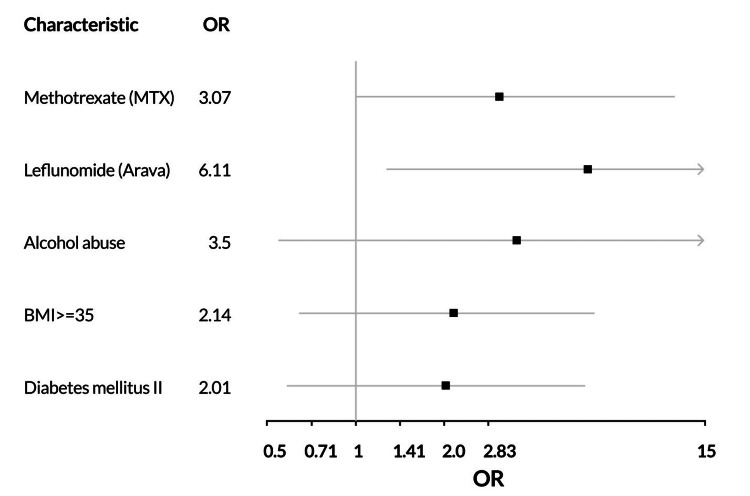
Forest plot for adjusted odds ratio (OR) for DMARD change or dose decrease due to liver abnormality. DMARD, disease-modifying anti-rheumatic drug.

Table 4 demonstrates the unadjusted odds ratios of clinical characteristics at the baseline visit in association with the primary outcome. Figure 1 details the adjusted odds ratios for the primary outcome. Leflunomide carried the highest odds ratio for the risk of DMARD change due to hepatotoxicity. Class II Obesity, Diabetes II, and alcohol abuse trended toward risk for the primary outcome, without statistical significance.

**Table 4:** Unadjusted odds ratios (ORs) for DMARD change or dose decrease due to liver abnormality. ^a^Included all the observed abnormalities except cirrhosis; ^b^Included all the observed abnormalities except cirrhosis other abnormalities. OR estimates, 95% CI, and p-values from exact logistic regression. P-values less than 0.05 were considered statistically significant. HDL, high-density lipoprotein; LDL, low-density lipoprotein; CCP, cyclic citrullinated peptide antibodies; RF, rheumatoid factor; DMARD, disease-modifying anti-rheumatic drug.

Characteristic	OR	95%CI	p-value
Male	0.91	(0.23, 2.67)	0.878
Age	1.01	(0.98, 1.05)	0.49
BMI≥35	3.15	(1.09, 8.38)	0.035
Diabetes mellitus II	2.75	(1.02, 6.88)	0.045
HDL<=40 mg/dL	2.98	(0.74, 12.0)	0.119
LDL>=160 mg/dL	1.19	(0.31, 5.25)	0.805
Triglycerides>=200 mg/dL	2.64	(0.46, 11.1)	0.243
Metabolic syndrome	1.52	(0.34, 14.4)	0.62
Alcohol abuse	3.19	(0.59, 11.8)	0.156
Current statin	1.72	(0.62, 4.36)	0.284
CCP or RF positive	0.89	(0.37, 2.26)	0.796
Abnormal liver Def 1^a^	1.19	(0.35, 4.47)	0.782
Abnormal liver Def 2^b^	1.58	(0.47, 5.53)	0.457
DMARD			
Methotrexate (MTX)	2.88	(1.07, 9.49)	0.035
Leflunomide (Arava)	3.14	(0.78, 9.79)	0.1
Hydroxychloroquine	0.64	(0.23, 1.58)	0.338
Sulfasalazine	0.9	(0.18, 2.99)	0.882

## Discussion

Our study does suggest that several surrogates of metabolic syndrome such as BMI and diabetes mellitus are likely risk factors for the development of DMARD hepatotoxicity. Particularly in patients that have no risk factors, leflunomide especially seems to carry a significant and independent risk of hepatotoxicity. While methotrexate trended to have some significance toward the primary outcome, the confidence intervals were too wide in our study to make a definitive assessment.

Several studies in the last three decades have suggested against the implication of methotrexate in chronic hepatotoxicity, or implicating methotrexate primarily in patients with a co-existing liver insult [5,6,9]. A recent study further confirmed that metabolic factors are much more likely than methotrexate to lead to long-term liver fibrosis, implying that the risk attributed to methotrexate historically has been overestimated [10].

Even after adjustment, leflunomide seemed to carry the highest risk for DMARD change due to liver toxicity in our study. This finding is consistent with prior prospective trials that showed slightly higher risks of hepatotoxicity with leflunomide compared to methotrexate and placebo [4]. Importantly, some cases of leflunomide liver injury can be idiosyncratic and severe and our study was not fully powered to isolate these fulminant cases.

Strengths of our study include a practical approach to how DMARDs are stopped for hepatotoxicity. Prior studies have implicated how provider perception of hepatotoxicity largely influences choice and withdrawal [11]. Strict guidelines on exactly when DMARDs are held due to transaminitis are lacking, so it is an interesting finding in our study that about two to three times the upper limit of normal is the practical threshold at which most providers stop a possibly hepatotoxic DMARD. Another strength of our study is the focus on rheumatoid arthritis in a diverse academic medical center. Many previous studies have suggested that psoriasis is associated with nonalcoholic fatty liver disease. Our study’s look at only rheumatoid arthritis eliminated extraneous rheumatic diseases from further confounding the picture. Finally, we also looked at numerous surrogate markers for metabolic syndrome which are important and practical tests for a typical clinician.

Several weaknesses were also present in our study. As a retrospective cohort study, the study is amenable to confounding bias. In addition, it is possible that the threshold of transaminitis at which methotrexate or leflunomide (historically drugs that have been purported to be hepatotoxic) were changed is higher than the threshold for which providers did so for sulfasalazine and other DMARDs. In addition, patients who were at high pre-existing risk for hepatotoxicity might have been pre-selected to be on a less hepatotoxic DMARD. Our study also lacked radiographic, ultrasound, or Fibroscan assessments of liver function which are more precise in their approach to long-term impacts on liver synthesis and function [9,10]. Finally, our study was conducted at a time when there was less use of biologics than currently and this may deter from a more full application to clinical practice.

Our study also had several additional limitations. The risk of hepatotoxicity may have been confounded by a number of additional medications including anti-inflammatory medications, diabetic medications, anti-hypertensive medications, and psychotropic medications which can intermittently lead to hepatotoxicity in some patients [12]. Also, rates of biologics use were relatively low in the study population. However, biologics have been found to have a variable impact on hepatotoxicity with most not being discretely associated with hepatotoxicity [13,14]. Our study is also not generalizable among other populations at present. Though the prevalence of liver disease is likely higher in populations with psoriatic arthritis, however, some studies do not necessarily suggest that the overall risk of DMARD hepatotoxicity is higher [15].

Though the study was underpowered for statistical significance, clinicians should consider features of metabolic syndrome as potential risk factors for hepatotoxicity with DMARD use. Further studies need to be performed to examine this relationship.

## Conclusions

The risk of hepatotoxicity with DMARDs (such as methotrexate and leflunomide) is likely influenced by pre-existing metabolic syndrome. Our study was underpowered to assess the risk of some of these features of metabolic syndrome, however, it suggests that the trend is toward serving as a risk factor for DMARD hepatotoxicity. Further studies should be performed on a larger scale to investigate these risk factors further.

## References

[1] Bath RK, Brar NK, Forouhar FA, Wu GY (2014). A review of methotrexate-associated hepatotoxicity. J Dig Dis.

[2] Kremer JM, Lee RG, Tolman KG (1989). Liver histology in rheumatoid arthritis patients receiving long-term methotrexate therapy. A prospective study with baseline and sequential biopsy samples. Arthritis Rheum.

[3] Candia R, Ruiz A, Torres-Robles R, Chávez-Tapia N, Méndez-Sánchez N, Arrese M (2015). Risk of non-alcoholic fatty liver disease in patients with psoriasis: a systematic review and meta-analysis. J Eur Acad Dermatol Venereol.

[4] Strand V, Cohen S, Schiff M (1999). Treatment of active rheumatoid arthritis with leflunomide compared with placebo and methotrexate. Leflunomide Rheumatoid Arthritis Investigators Group. Arch Intern Med.

[5] Sheth SG, Gordon FD, Chopra S (1997). Nonalcoholic steatohepatitis. Ann Intern Med.

[6] Hamaguchi M, Kojima T, Takeda N (2005). The metabolic syndrome as a predictor of nonalcoholic fatty liver disease. Ann Intern Med.

[7] Lertnawapan R, Chonprasertsuk S, Siramolpiwat S (2019). Association between cumulative methotrexate dose, non-invasive scoring system and hepatic fibrosis detected by Fibroscan in rheumatoid arthritis patients receiving methotrexate. Int J Rheum Dis.

[8] Langman G, Hall PM, Todd G (2001). Role of non-alcoholic steatohepatitis in methotrexate-induced liver injury. J Gastroenterol Hepatol.

[9] Atallah E, Grove JI, Crooks C (2023). Risk of liver fibrosis associated with long-term methotrexate therapy may be overestimated. J Hepatol.

[10] Sandrin L, Fourquet B, Hasquenoph JM (2003). Transient elastography: a new noninvasive method for assessment of hepatic fibrosis. Ultrasound Med Biol.

[11] Mittal N, Sharma A, Jose V, Mittal R, Wanchu A, Bambery P (2012). Causes of DMARD withdrawal following ADR within 6 months of initiation among Indian rheumatoid arthritis patients. Rheumatol Int.

[12] Chitturi S, George J (2002). Hepatotoxicity of commonly used drugs: nonsteroidal anti-inflammatory drugs, antihypertensives, antidiabetic agents, anticonvulsants, lipid-lowering agents, psychotropic drugs. Semin Liver Dis.

[13] Aithal GP (2011). Hepatotoxicity related to antirheumatic drugs. Nat Rev Rheumatol.

[14] French JB, Bonacini M, Ghabril M, Foureau D, Bonkovsky HL (2016). Hepatotoxicity associated with the use of anti-TNF-α agents. Drug Saf.

[15] Amital H, Arnson Y, Chodick G, Shalev V (2009). Hepatotoxicity rates do not differ in patients with rheumatoid arthritis and psoriasis treated with methotrexate. Rheumatology (Oxford).

